# Spinal cord injury and electrical stimulation: analysis of neuroplasticity in a case report

**DOI:** 10.3389/fresc.2025.1557010

**Published:** 2025-06-19

**Authors:** Orcizo Francisco Silvestre, Julia Silva e Lima Schleder, Bruna Valentina Zuchatti, Cintia Kelly Bittar, Carla Alves Fakih, Marina Squarizi Simões Chagas, Vinicius Taboni Lisboa, Alberto Cliquet Junior

**Affiliations:** 1 Faculty of Medical Sciences, UNICAMP- State University of Campinas, Campinas, Brazil; 2 Faculty of Nursing, UNICAMP- State University of Campinas, Campinas, Brazil; 3 Orthopedic Department, Pontifical Catholic University of Campinas (PUC), Campinas, Brazil

**Keywords:** spinal cord injury, electrical stimulation, post-acute COVID-19 syndrome, trauma, neuroplasticity

## Abstract

**Introduction:**

Spinal Cord Injury (SCI) is a highly prevalent condition, with just below 1 million new cases yearly, deriving for traumatic and non-traumatic causes. It is a significant cause for disability, greatly impacting quality of life of affected individuals, and as such, requires effective rehabilitation methods in order to maintain daily function. Neuromuscular Electrical Stimulation (NMES) is a helpful treatment, stimulating muscle contraction and plasticity through electrical currents.

**Methods:**

This is a Case-Report of two cases with different SCI causes, submitted to a 1-year treatment with NMES under identical protocols. ASIA neurological examination with AIS classification was performed before and after treatment, as well as surface Electromyographic assessment for the Vastus Lateralis and Rectus Femoris muscles bilaterally.

**Results:**

Neurological recovery was remarkable after 1 year, with AIS increasing from a score of A to C in the first case and B to C in the second. EMG assessment showed a bilateral increase of peak values as well as successful Quadriceps muscle contraction generating knee extension.

**Conclusion:**

Neuromuscular electrical stimulation may be a promising strategy in the rehabilitation of spinal cord injuries, with the potential to aid in functional recovery and modulation of neuroplasticity. Preliminary observations, such as those in this case report, suggest that the technique may be associated with improvements in mobility and quality of life in patients, although controlled studies are needed to confirm these effects.

## Introduction

Spinal Cord Injury (SCI) is prevalent in 20.6 million people globally and has a 0.9 million yearly incidence rate. Causes range from traumatic to non-traumatic (1). The most significant of traumatic causes being neurotrauma, a combination of traumatic brain injury (TBI) and spinal cord trauma (2). Non-traumatic causes can derive from neoplasms, degenerative conditions, iatrogenic, infectious, idiopathic, vascular and autoimmune diseases (3). SCI frequently causes motor and sensory losses that directly or indirectly impact the quality of life of affected individuals (1).

**Figure 1:**
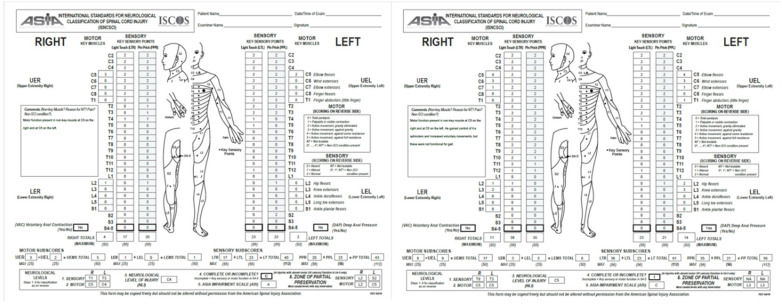
ASIA assessment sheet for case 1 before (left) and after (right) one year rehabilitation treatment with NMES.

**Figure 2:**
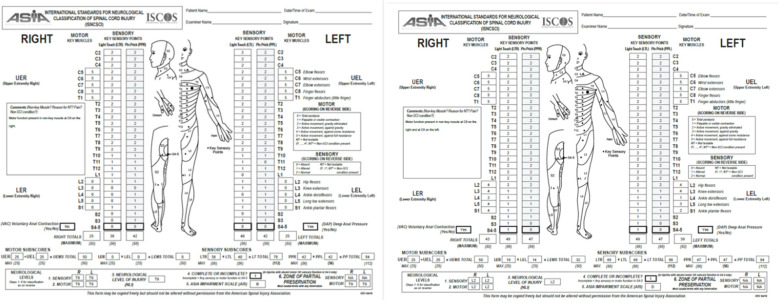
ASIA assessment sheet for case 2 before (left) and after (right) one year rehabilitation treatment with NMES.

**Figure 3:**
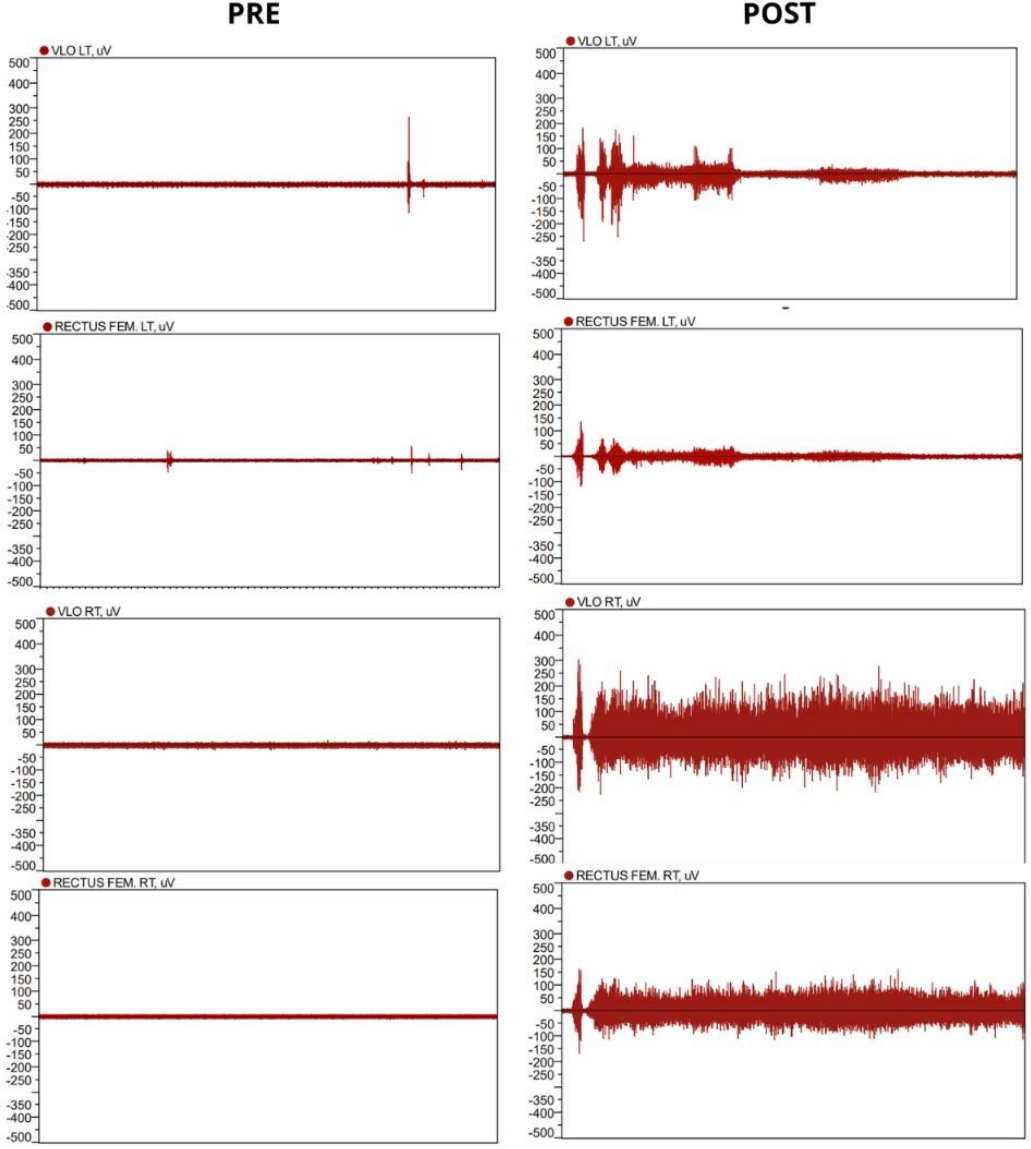
First case surface EMG at the beginning (left) and one year after initial NMES treatment (right).

**Figure 4:**
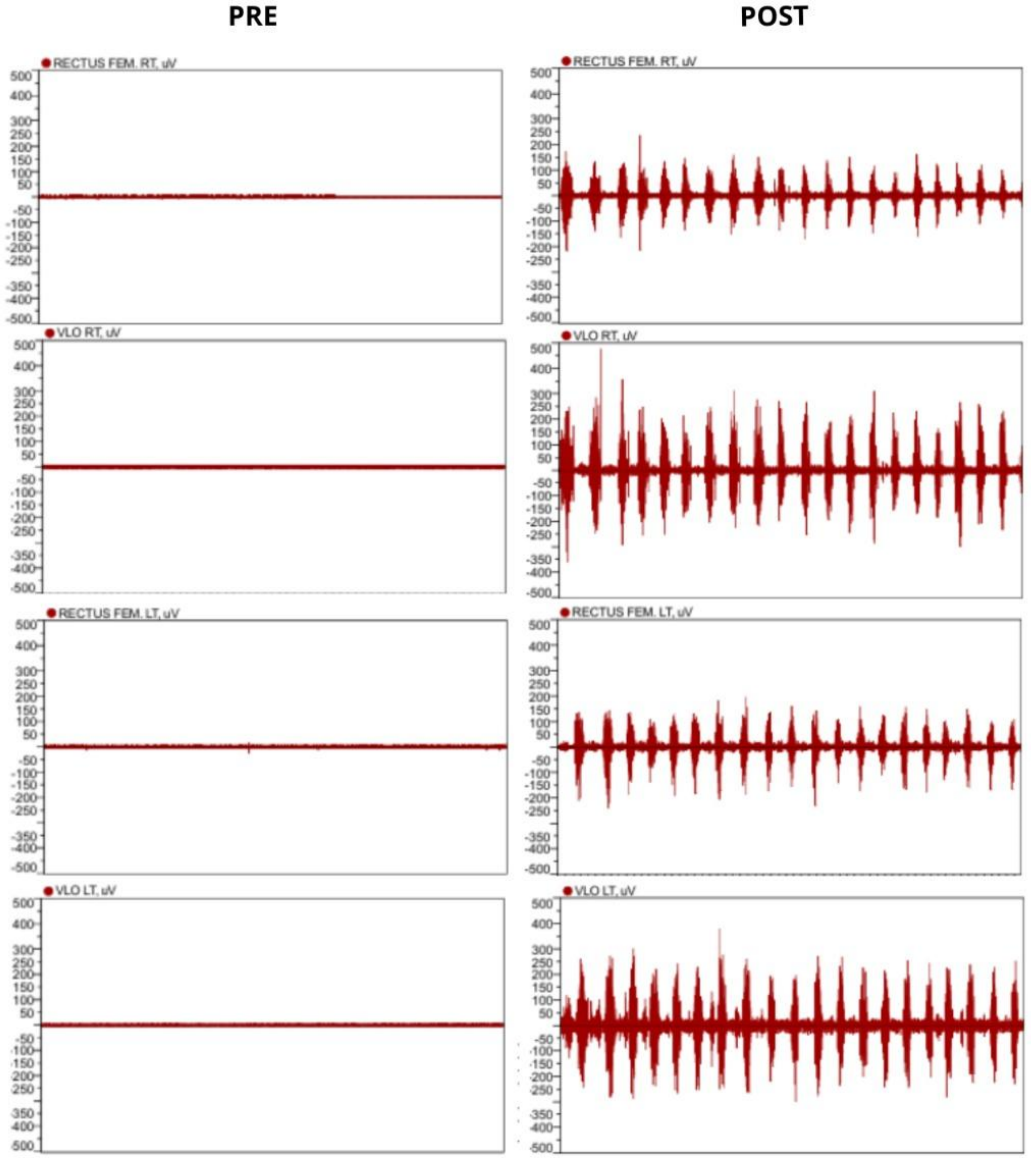
Second case surface EMG at the beginning (left) and 1 year after initial NMES treatment (right).

As such, an effective rehabilitation treatment for SCI is extremely important, with neuromuscular electrical stimulation (NMES) being a current efficacious option. NMES is the application of a low frequency electrical current to specific muscle groups in order to obtain muscle contraction and perform neural activation (4). According to Silvestre et al. (5), NMES can generate a possible gain in neuroplasticity, which was observed through electromyographic assessment, and consequently help to gain some motor function. NMES promotes artificial movement initially, followed by neuroplasticity and independent movement. The objective is to promote functional independence and help with activities such as walking, standing and reaching and/or manipulating objects (6).

This study aims to present the contribution of Neuromuscular Electrical Stimulation in two patients with different causes of SCI, one traumatic and one non-traumatic (post-COVID-19 Syndrome).

## Methods

This is a case study that describes and analyzes the application of NMES in two patients with spinal cord injury, one secondary to a motor vehicle accident causing neurotrauma and one post-COVID-19 Syndrome. The protocols used, the therapeutic responses observed, and the results achieved are highlighted, seeking comprehension of the impact of this intervention in two different specific contexts.

This study is descriptive and longitudinal and covers three main stages: initial assessment, treatment with electrostimulation and follow-up.

Initial evaluation consisted of a complete clinical examination, application of the American Spinal Injury Association (ASIA) neurological exam with AIS classification and one-minute NORAXON® system surface electromyographic (EMG) assessment, occuring before the beginning of treatment protocols. During EMG, participants were asked to perform repeated knee extensions for 60 s. Data was collected for Vastus Lateralis and Rectus Femoris muscles, following predetermined laboratory protocols.

A four-channel electro stimulator was used for the second stage, the rehabilitation treatment stage, which had a single-phase signal of 25 Hz and a duration of 300 μs, with an intensity of 70–150 V with rectangular pulses. Sessions were held twice a week, with an average duration of 30–40 min each, during which time the Quadriceps and Anterior Tibial muscles would be cyclically stimulated every 10 s, generating knee extension and ankle dorsiflexion. No practiced walking with support was performed during this described period.

Post-treatment follow-up consisted of evaluation of patients after 1 year of treatment with full neurological clinical examination, ASIA exam application with AIS classification and one-minute NORAXON® system surface electromyography (EMG) system were performed to assess clinical parameters.

The data collected through the neurological examination were compiled into an Excel® spreadsheet and descriptive statistics were performed through graphs and images, in which the neurological change of the cases is presented. In addition, Noraxon® hardware and software were used to capture electromyography signals in real time.

## Ethical aspects

The present study was approved by the Research Ethics Committee of the State University of Campinas (CEP—UNICAMP), under the approval number 5758947 of November 16, 2022, in accordance with Resolution 466/2012 of the Brazilian National Health Council.

## Case reports

### First case

54-year-old male, victim of a vehicular accident in 1998. Patient was diagnosed with a C5-C6 level SCI at the time of the event, as well as TBI, with partial loss of brain mass and skull bone coverage. The participant previously received medical monitoring and physical therapy, exclusively in the form of passive range of motion exercises, which was the only form of rehabilitation treatment tolerated at the time. NMES treatment began in January 2019. In early 2020 rehabilitation was interrupted due to the COVID-19 pandemic and was resumed in August 2021.

At initial clinical examination, in the outpatient rehabilitation clinic, the participant had present contraction of elbow flexors bilaterally, with Medical Research Council (MRC) muscle strength scale (7) grade of 2 on the left side and 3 on the right side. Remaining motor function was only present for hip flexors on the right side, with a MRC grade of 1. Sensory function was present at dermatome T1 and above. He had no Voluntary Anal Contraction (VAC) or Deep Anal Pressure (DAP) sensation on the first exam.

NMES treatment was performed according to previously described protocol. Sessions were performed twice weekly between mid-January and mid-December, with approximately 96 sessions of NMES per year.

The ASIA exam with AIS classification was initially conducted before the start of treatment and then after 1 year. EMG was also performed on the Rectus Femoris and Vastus Lateralis muscles at the start and after 1 year of treatment.

### Second case

72-year-old male patient who contracted COVID-19 in 2021. Within 1 month post-COVID-19 he began loss of lower limb motricity and diffuse lower limb spasticity. Thorough investigation was performed to exclude other causes of medullary lesion and the patient was diagnosed with T9 level SCI secondary to post-COVID-19 Syndrome in 2022.

At initial clinical examination, in the outpatient rehabilitation clinic, sensory function was preserved above dermatome T9 bilaterally. The participant presented normal upper extremity motor function, and MRC (7) grade of 2 for hip flexors on the left side and 3 on the right. Knee extensor function was graded 4 bilaterally. He presented no Voluntary Anal Contraction (VAC) but preserved Deep Anal Pressure (DAP) sensation on the first exam.

The patient began rehabilitation treatment at this Hospital in August 2022, directly with electrostimulation. Treatment with NMES was performed on the quadriceps muscles for knee extension. He performed an average of 96 NMES sessions per year, ASIA with AIS classification and surface EMG in the Rectus Femoris and Vastus Lateralis muscles were assessed after the end.

## Results

During NMES treatment, the ASIA neurological assessment was performed in order to monitor the neurological evolution of the participants, which is a gold standard assessment scale to identify the extent of the level of spinal cord impairment (8). It was also observed, through examination, that both patients treated with NMES presented significant neurological improvements during treatment. The images below show the evolution of cases 1 and 2.

For case 1, the participant presented with an AIS A on the start of treatment and B after one year, with neurological level C5 on both assessments. For case 2, the participant presented with and AIS B on arrival and D after one year of treatment, with neurological level progressing from T9 to L2. Below are Figures 1–4.

## Discussion

The present case report describes the rehabilitation process of two cases with spinal cord injuries. The first, a SCI secondary to neurotrauma causing tetraplegia, and the second, secondary to post-COVID-19 syndrome, causing paraplegia.

Case 1 presented greater complexity due to the presence of irreversible sequelae caused by pyroptosis, secondary to the combination of SCI and TBI (9). It should be noted that SCI and TBI are complex conditions with limited therapeutic and rehabilitation resources, which highlights the significance of the results obtained. In case 2, SCI can be characterized as non-traumatic secondary to post-COVID-19 syndrome. Although the specific cause of the injury is still unknown, it may be due to a thromboembolic event (10). COVID-19 infections can cause thrombotic state, with a study by Cui (11) et al. showing the presence of thromboembolism in 25% of 81 patients with severe COVID-19 infections. This can cause infarction either directly by the obstruction of blood flow, or indirectly through renal dysfunction (12).

During ASIA assessment, it was also observed that case 1 gained some DAP sensory function and case 2 VAC motor function. These were not directly stimulated and although the role of NMES is unclear, further research may be useful in understanding this. However, studies have shown that residual neural pathways can remain present even in complete SCI, which may explain the evolution of both of these cases (14).

Electromyographic assessment showed important results in both cases as well. For case 1, there was muscle activation for one effective contraction of the Quadriceps muscle, although it was followed by a period of spasticity and subsequently, fatigue. Even so, the EMG results showed great improvement from the first evaluation, a year prior. For the second case, surface EMG showed effective cadenced movements for the entire minute of the evaluation, which evidenced that although muscle strength at the ASIA assessment did not change, after one year of NMES the subject became capable of sustaining repeated cadenced quadriceps contraction. Preliminary studies indicate that EMG can serve as a noninvasive tool to estimate muscle activation patterns in patients with SCI, but its clinical validity requires confirmation in larger and more diverse samples (6).

## Study limitation

A limitation of this study is the small sample size of patients included in the research. Small sample sizes may make it difficult to identify consistent patterns and increase the influence of individual variables, including the possibility that the results obtained may be significantly biased by the rehabilitation period or the natural evolution of the spinal cord injury. In addition, the lack of diversity in the sample, such as differences in injury severity or time post-injury, may limit the generalization of the findings to broader populations. Therefore, future studies with larger cohorts are needed to better evaluate the effects of electrical stimulation on neuroplasticity after spinal cord injury.

Although this study focused on neuromuscular electrical stimulation, other emerging stimulation modalities, such as electrical stimulation and epidural spinal cord stimulation, have been explored for post-spinal cord injury rehabilitation. While NMES predominantly acts on peripheral muscle activation and spinal reflexes, electrical stimulation modulates cortical excitability (15, 16) and epidural electrical stimulation targets residual spinal cord circuits (17). Our results, which showed the possible efficacy of NMES in sensory recovery, contrast with studies of other techniques, in which motor responses are more frequent (15, 16). This difference may reflect distinct mechanisms: NMES acts via afferent pathways (Ia/II), while epidural stimulation recruits dorsal fibers directly. Future studies combining these techniques could enhance synergies between central and peripheral plasticity.

## Conclusion

Neuromuscular electrical stimulation may be a promising strategy in the rehabilitation of spinal cord injuries, with the potential to aid in functional recovery and modulation of neuroplasticity. Preliminary observations, such as those in this case report, suggest that the technique may be associated with improvements in mobility and quality of life in patients, although controlled studies are needed to confirm these effects.

## Data Availability

The original contributions presented in the study are included in the article/Supplementary Material, further inquiries can be directed to the corresponding author.
