# Correction: Spinal cord injury and electrical stimulation: analysis of neuroplasticity in a case report

**DOI:** 10.3389/fresc.2026.1925781

**Published:** 2026-08-03

**Authors:** Orcizo Francisco Silvestre, Julia Silva e Lima Schleder, Bruna Valentina Zuchatti, Cintia Kelly Bittar, Carla Alves Fakih, Marina Squarizi Simões Chagas, Vinicius Taboni Lisboa, Alberto Cliquet Junior

**Affiliations:** 1 Faculty of Medical Sciences, UNICAMP- State University of Campinas, Campinas, Brazil; 2 Faculty of Nursing, UNICAMP- State University of Campinas, Campinas, Brazil; 3 Orthopedic Department, Pontifical Catholic University of Campinas (PUC), Campinas, Brazil

**Keywords:** spinal cord injury, electrical stimulation, post-acute COVID-19 syndrome, trauma, neuroplasticity

There was a mistake in the keywords list as published. The term “neuroplasicity” has been corrected to “neuroplasticity”.

There was a mistake in Figure 1 as published. There was an error in the completion of the table. The correct Figure 1 appears below:

There was a mistake in Figure 2 as published. There was an error in the completion of the table. The correct Figure 2 appears below:

A correction has been made to the Discussion, Paragraph 3, to reflect the corrected neurological level presented in Figures 1 and 2.

**Figure 1:**
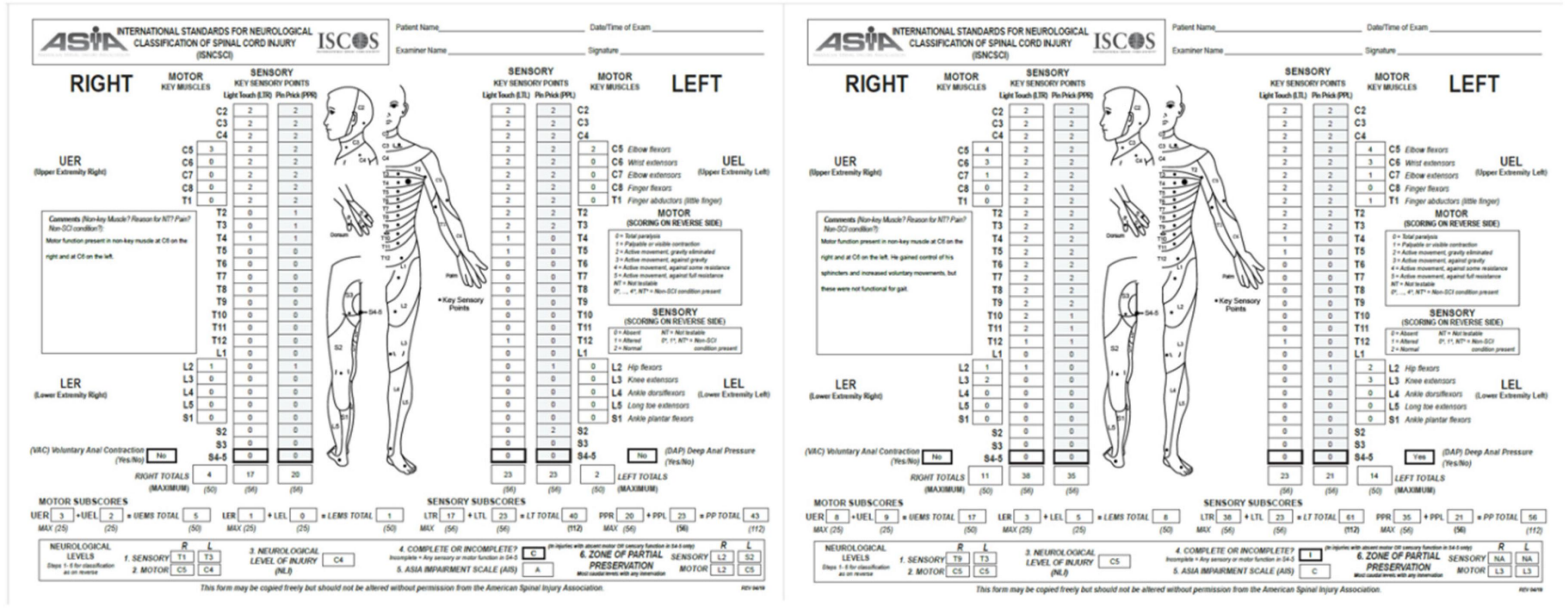
ASIA assessment sheet for case 1 before (left) and after (right) one year rehabilitation treatment with NMES.

**Figure 2:**
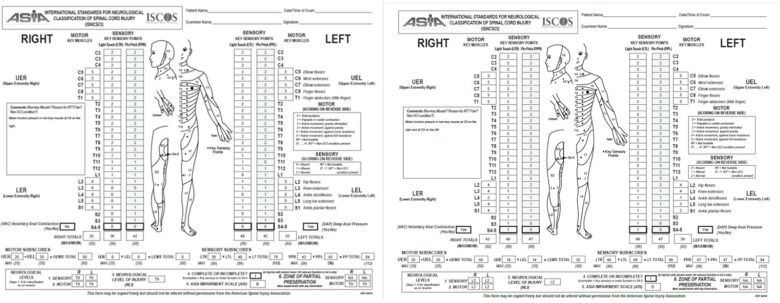
ASIA assessment sheet for case 2 before (left) and after (right) one year rehabilitation treatment with NMES.

This paragraph previously stated:

“NMES was effective in both sensory and motor changes, in both cases, in which it was the only form of rehabilitation being conducted at the time. For the first case, sensory level changed from a T1 on the right and T3 on the left to T9 bilaterally, and although motor level remained at C5 at the end of treatment, the subject gained lower limb motor function, especially knee extensor function. For the second case, sensory level changed from T9 to L2 bilaterally, and whilst there were no changes for knee extension during ASIA assessment, remaining motor lower limb functions showed improvement, although these were not directly stimulated. Previous studies (6, 13) suggest that NMES may improve sensory and motor functions, and similar findings were observed in this study. However, due to methodological limitations and sample size, it is not possible to establish a direct relationship.”

The corrected paragraph appears below:

“NMES was effective in both sensory and motor changes, in both cases, in which it was the only form of rehabilitation being conducted at the time. For the first case, the sensory level changed from T1 on the right and T3 on the left at baseline to T9 on the right and T3 on the left after treatment. The participant improved neurologically from C4 AIS A to C5 AIS C and recovered motor function in the lower limbs, particularly knee extensor function. Although the final motor level was C5 bilaterally, gains in lower extremity motor function were observed. For the second case, the sensory level changed from T9 to L2 bilaterally. In addition, lower extremity motor function improved, including a marked recovery of knee extensor strength, which increased from 0/5 at baseline to 4/5 bilaterally at the final evaluation. Improvements were also observed in the other lower limb motor functions. Previous studies (6, 13) suggest that NMES may improve sensory and motor functions, and similar findings were observed in this study. However, due to methodological limitations and sample size, it is not possible to establish a direct relationship.”

The original version of this article has been updated.

